# Brexit and trade policy: an analysis of the governance of UK trade policy and what it means for health and social justice

**DOI:** 10.1186/s12992-021-00697-1

**Published:** 2021-06-09

**Authors:** May C. I. van Schalkwyk, Pepita Barlow, Gabriel Siles-Brügge, Holly Jarman, Tamara Hervey, Martin McKee

**Affiliations:** 1 Faculty of Public Health and Policy, London School of Hygiene and Tropical Medicine, 15-17 Tavistock Place, London, WC1H 9SH UK; 2 Department of Health Policy, London School of Economics, London, UK; 3 Department of Politics and International Studies, University of Warwick, Coventry, UK; 4 Department of Health Management and Policy, University of Michigan School of Public Health, Ann Arbor, MI USA; 5 The City Law School, City, University of London, London, UK

**Keywords:** Population health, Political determinants of health, Health policy, Social justice, Democracy, Trade policy, Governance, Brexit, Transparency, Participation

## Abstract

**Background:**

There is an extensive body of research demonstrating that trade and globalisation can have wide-ranging implications for health. Robust governance is key to ensuring that health, social justice and sustainability are key considerations within trade policy, and that health risks from trade are effectively mitigated and benefits are maximised. The UK’s departure from the EU provides a rare opportunity to examine a context where trade governance arrangements are being created anew, and to explore the consequences of governance choices and structures for health and social justice. Despite its importance to public health, there has been no systematic analysis of the implications of UK trade policy governance. We therefore conducted an analysis of the governance of the UK’s trade policy from a public health and social justice perspective.

**Results:**

Several arrangements required for good governance appear to have been implemented – information provision, public consultation, accountability to Parliament, and strengthening of civil service capacity. However, our detailed analyses of these pillars of governance identified significant weaknesses in each of these areas.

**Conclusion:**

The establishment of a new trade policy agenda calls for robust systems of governance. However, our analysis demonstrates that, despite decades of mounting evidence on the health and equity impacts of trade and the importance of strong systems of governance, the UK government has largely ignored this evidence and failed to galvanise the opportunity to include public health and equity considerations and strengthen democratic involvement in trade policy. This underscores the point that the evidence alone will not guarantee that health and justice are prioritised. Rather, we need strong systems of governance everywhere that can help seize the health benefits of international trade and minimise its detrimental impacts. A failure to strengthen governance risks poor policy design and implementation, with unintended and inequitable distribution of harms, and ‘on-paper’ commitments to health, social justice, and democracy unfulfilled in practice. Although the detailed findings relate to the situation in the UK, the issues raised are, we believe, of wider relevance for those with an interest of governing for health in the area of international trade.

## Background

Our understanding of the determinants of health has moved progressively upstream. The immediate causes of disease, such as micro-organisms and carcinogens, have long been recognised as acting within a social milieu. More recently, scholars have revisited early thinking on the global political determinants [1] and, over the past few decades, the commercial determinants of health, recognising the growth in power of multi-national corporations and the globalisation of their impacts [2, 3]. Research on trade policy lies at the intersection of these two fields, and a growing body of research has identified its implications for health and social justice [4, 5]. Much of this has focused on how trade liberalisation and trans-national commerce has increased consumption of harmful products, in particular energy-dense food and beverages [6, 7]. Some work has also examined the benefits of trade through increased economic growth, employment, and food security, finding that the benefits are often distributed unevenly within and between societies [8–10]. Other work has looked at how the tobacco industry has, albeit often unsuccessfully, sought to use international trade agreements to overcome tobacco control policies [11, 12]. More generally, literature has also focused on the constraints imposed by trade and investment agreements on countries’ policy flexibility in the area of public health [13–15]. These examples, and others, have led some to question why public health is, too often, excluded from these discussions, and to advocate for reforms to trade policy governance, particularly the conduct of trade negotiations [16–19].

### The United Kingdom context

The United Kingdom’s (UK’s) decision to leave the European Union (EU) brought these issues to the fore, generating a heated debate [20]. While different views about international trade can be found in most countries, the prominence of Brexit in domestic political discourse in the UK, coupled with the polarisation of views, has made this debate unusually visible. Perhaps the only thing that all sides can agree on is that trade policy arrangements have changed and will continue to change. However, some see this as an opportunity for health whilst others highlight several threats. One product has come to symbolise this division: chlorine-washed chicken. Some view the existing ban on its import into the EU as an unnecessary barrier to a product that is cheap and safe [21], while others view it as a necessary safeguard against food produced in unhygienic conditions that disregard animal welfare [22–24].

The UK is arguably unique in being a major global economy that lacks the trade expertise seen in other major economies, as negotiations have been undertaken on its behalf by the EU for four decades [25]. This creates substantial challenges as the UK seeks to negotiate ambitious agreements with much more powerful countries, including the US, with more experienced trade negotiating machineries. As set out below, these trade deals risk compromising health if governance structures are inadequate.

### Trade policy governance, health, and social justice

Who should decide and how, when health and social justice issues arise during trade negotiations? Who decides what items on the agenda are prioritized: health, equity, and environmental considerations, or market access and corporate profits? The answers to these questions are heavily influenced by trade policy governance. Governance guides trade policy agendas and who decides the agenda, in turn influencing who serves to benefit from trade, who is at risk of harm or loss, and what measures are taken to ensure an equitable distribution of benefits and mitigation of harms in ways that prioritise the most vulnerable in society. Trade policy governance therefore has implications for health, health equity, and other social justice considerations, such as the equitable distribution of wealth and opportunities, including opportunities to exercise democratic voice, within societies.

For example, health equity constitutes social justice with respect to health, and is the absence of systematic, unnecessary, and avoidable differences in health, both within and between countries [26, 27]. In being unnecessary and avoidable in nature, health inequities are regarded as unfair and unjust, and from a normative perspective, it is expected that reasonable action be taken to mitigate for, or address, health inequities and other inequitable distributions of power, resources and opportunities. Whether or not such actions can be taken and whether they are just depends critically on whether governance structures provide scope for any harms to health from trade deals to be identified, through transparent publication of negotiation mandates; for mitigating actions to be put forward by those with relevant expertise, via public consultations; and whether electorates have an opportunity to vocalise opposition and hold politicians to account for any harms to health they consider unjust.

Yet, civil society groups, health advocates, and scholars have long noted that trade negotiations and governance typically provide insufficient scope for health issues to be taken into consideration [28–30]. Particular worries have become pronounced among the public health community in the UK, given some of the stated aims of US trade negotiators in areas such as agriculture and pharmaceuticals that they see as posing a potential threat to health [31]. The UK has had to start almost from scratch, creating a new government ministry, the Department for International Trade (DIT), in July 2016 [32]. However, the DIT must not only negotiate new trade deals. It has taken on responsibility for various existing bodies working in, for example, support of British exports and foreign investment [33]. The DIT has also retaken the competence to negotiate International Investment Agreements (IIAs) covering, amongst other things, the protection of foreign investors. The DIT’s work was, and continues to be, scrutinised by another new body, the House of Commons International Trade Committee (ITC), charged with the task of examining “the expenditure, administration and policy of the Department for International Trade and its associated public bodies” [34]. Other Parliamentary Committees also scrutinise the work of the DIT, but do not have a remit focused specifically on the work of the DIT.

There are concerns about the degree of public and parliamentary participation, and the phenomenal external burden placed on the UK by the COVID-19 global pandemic. These concerns have led to calls from civil society advocates for trade agreement negotiations to be postponed [35, 36], but these have nevertheless continued apace. The role of the devolved nations/administrations and the degree of participation they are afforded, especially in the negotiation of international trade agreements, is also of particular concern given the significant stake they have in the UK’s future trade policy [37].

Systems of governance guide decision-making and policy implementation; influencing *which* policies are adopted, *who* participate and decides, and *how* they are implemented and monitored [38]. Strong governance systems are essential to achieving policy aims, avoiding corruption, anticipating and mitigating unintended consequences, and are critical to ensuring that decisions are made in ways that adequately consider the health and equity implications [38]. Whose interests will be prioritised and who decides depends in part on the trade policy structures that have been established and how they are governed. To understand the health and equity implications of the UK’s post-Brexit trade agenda, and how and if these impacts will be considered, it is important to scrutinise trade policy governance [17].

Despite its importance to public health, particularly in contested areas such as trade [17], there has been no systematic analysis of the implications of UK trade policy governance for public health. We have therefore conducted an analysis of the governance of the UK’s trade policy, from the inception of the DIT and ITC through to November 2019, examining the work of the DIT, drawing on the investigations that have been undertaken by the ITC, with an eye on what this could mean for public health and social justice. Although the detailed findings relate to the situation in the UK, the issues raised are, we believe, of wider relevance for those with an interest in governing for health in the area of international trade.

## Results

**Fig. 1:**
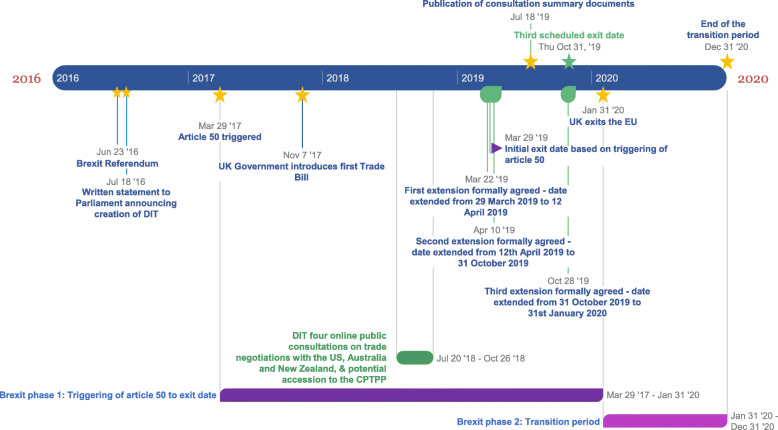
Brexit and UK Trade policy - Timeline of key events

Our analysis identified a number of important considerations relevant to UK trade policy governance, using publicly available data generated by the DIT and ITC. Here we present our main findings, illustrating the breadth and nature of the governance issues that have arisen. We highlight key findings on potential strengths and weaknesses in trade policy governance that are likely to have particular relevance for health as the UK seeks to establish itself as an independent trading partner outside of the EU. We report each of them under the individual TAPIC framework elements (transparency, accountability, participation, integrity, capacity) [38]. In addition, to provide context, Fig. 1 displays a timeline of key events.

### Transparency

An institution is said to be transparent when decisions, and the processes and grounds on which decisions are made, can be observed and the public and other relevant actors are informed about them [17, 38, 39]. Transparency is more than simply recording activities but requires that the information about them is readily accessible, accurate, timely, and comprehensive, and is presented in a way that is comprehensible, taking account of the technical issues that might arise [38]. Transparency is central to empowering members of health, environmental and social justice communities including health professionals, civil society and researchers, for example, to understand *how* decisions are being made, *what* is being considered and *why*, and to identify potential impacts. In the UK context, transparency between central government and the devolved nations/administrations is critical to facilitating the involvement of the latter, which is key to the establishment and implementation of inclusive and coherent trade policy [37]. Our analysis identified actions and structures that may either promote or compromise transparency.

Both the DIT and ITC publish a large amount of information on their websites and both have active social media accounts. For example, the News and communications section of the DIT website contains pronouncements about trade and investment promotion activities, consultations, ministerial speeches, press releases, and trade-related legislation, all disseminated in a timely manner. Other sections of the website report policy papers and consultations, research and statistics, and information that has been released under Freedom of Information legislation. The DIT governance section lists the membership of its main decision-making, executive, and managerial bodies, including the Department’s board, chaired by the Secretary of State, its Executive Committee, and its Audit and Risk Assurance Committee [40]. Formal hearings of the ITC are broadcast on the Parliament TV channel, which permits clips to be copied and shared, for example on social media, and are also archived. Submissions and other relevant material are also published on its website [41]. The ITC News section is updated in a timely manner, listing inquiries that the Committee is planning to hold and updates on those that are ongoing or completed. This includes the aim and purpose of the inquiry, what the Committee intends to cover in each session, and the witnesses it intends to question. It contains web links to recordings and transcripts of hearings. Summaries of Committee hearings are published, with explanatory notes and quotations from the Committee chair.

There were, however, other processes that were less transparent. For example, while the DIT reported on meetings held by the US-UK Trade and Investment Working Group, including a brief description of topics covered, it did not publish detailed agendas and minutes. It has only been possible to ascertain this material from leaked documents [42]. A second example involved the Department’s Strategic Trade Advisory Group (STAG), which brings together representatives of business, trade unions, and civil society, with its stated aim to “discuss trade policy” and “ensure trade policy is informed by a broad range of views” [43]. Its membership is published, but members are required to sign nondisclosure agreements. Only summaries of its meetings, and not transcripts, are published [43] so it is difficult to ascertain the evidence used in formulating its advice and the processes by which different perspectives are reconciled. The Department publishes a lengthy annual report [44], including a performance register, but it was not clear how this performance had been assessed, nor did we find any recognition of criticism of the DIT’s work by the ITC relating to lack of preparedness or adherence to timelines (discussed later). The DIT has also engaged in several public consultations and, accompanying each, it provided support materials outlining what it sees as relevant evidence, including statistics on trade and explanations of specialist terms [45]. However, it was notable that these accompanying texts focused predominantly on the benefits of increased trade. We found no evidence of how potential risks to health, the wider determinants of health, or the inequitable distribution of potential detrimental (or beneficial) impacts have been or will be taken into consideration. There was also some information that appeared misleading. For example, in a press release announcing publication of summary reports of the consultation outcomes, it was reported that the Department had also conducted nationwide market research indicating that two thirds of the UK public support free trade agreements and only 3% are opposed to them [46]. While technically correct, this failed to mention that the survey also revealed a low level of public understanding of trade [47].

Our examination of the proceedings of the ITC pointed to several concerns about transparency by ministers. The ITC has been concerned about the lack of clear and timely guidance provided during the Brexit negotiations. For example, its Chair expressed surprise that the government only published the UK’s tariff regime, that would be adopted in the event of ‘no deal’ with the EU, 16 days before the planned Brexit date. The Chair remarked that “It’s unforgivable that businesses have been deprived of this crucial information until this late hour” [48]. Later in 2019 (after two extensions to the Brexit proceedings had been granted), following a session on the impact of a ‘no deal’ scenario on trade with third (non-EU) countries the Chair commented that “The faces at the top of the Government may have changed, but the lack of clarity for businesses attempting to plan for non-EU trade following a no-deal Brexit remains a matter of considerable concern” [49]. When examining the potential impacts of tariffs that might be imposed in the event of a ‘no deal’ Brexit, the Committee Chair stated that “Not providing such important information is either a concerning oversight or, more concerningly, an intentional omission … I look forward to receiving Ms Truss’s explanation of why her Department is not setting out the full facts, and to hearing whether she intends to rectify this information gap in the future” [50]. Although the Government agreed to address this issue, the Committee Chair noted that they “should not have had to ask for it in the first place” [51].

There were other areas in which the ITC identified what it considered a lack of transparency. One was the unwillingness of the DIT to be clear about what was realistically feasible within the time it had to complete negotiations on World Trade Organisation (WTO) schedules and the rollover of EU trade agreements, and establish a functioning Trade Remedies Authority. The Committee repeatedly questioned assurances from the DIT that its work was progressing on schedule, deadlines would be met, and policies and systems would be in place to protect businesses, workers, and consumers. This led the ITC to advise the government in March 2018 to develop a risk registry [52]. But this was not established. The Committee also questioned the transparency of data reported by the DIT on Foreign Direct Investment, suggesting that it was cherry picking [53]. The ITC and expert witnesses raised concerns about lack of detail and use of vague language by the Government regarding its proposed strategies and intended future actions [54–56], which hinders transparency and scrutiny.

The Committee’s concerns about transparency were sufficient to lead it to conduct a specific inquiry on the topic [55]. When launching the final report in 2018, the Committee chair said “Current Government plans for the transparency and scrutiny of future trade negotiations are characteristically vague and attempt to dress poor planning up as pragmatism” [54]. The Committee recommended that the government should “operate from a presumption of transparency” [54], noting how expert witnesses had testified as to how a transparent and inclusive approach would strengthen the UK’s negotiating position [55]. They also commented on the value of transparency as a tool to reduce the spread of incorrect information, which was widespread in some highly contested areas, such as Investor-to-State Dispute Settlement mechanisms,^1^ and with regard to the impact of any negotiations on the NHS. In this respect they argued that a lack of transparency fuels mistrust and while secrecy may sometimes be justified, openness should be the default position [55].

Their conclusion resonated with evidence given by witnesses who commented on the culture of secrecy that they saw as characterising the British government’s approach, which they contrasted with the more open approach taken by other governments. This was seen by some as reflecting how trade had become a party political issue, with exclusion of opposition politicians from discussions. As one witness noted “… it is really important that individual trade deals are not seen as a party political issue. Governments may change and what a waste of several years’ negotiation if suddenly the other party comes and always rejects it. It is a waste and it does not take us forward. The idea of trade deals should be that they benefit the country as a whole. There should not be Conservative trade deals and Labour trade deals. There should just be good trade deals for the country” [57]. Based on their inquiry, the Committee recommended that certain elements of consultation and oversight should be made statutory and called for the level of transparency that applies to EU trade negotiations not to be reduced after exiting the EU. In its 2015 ‘Trade for All’ strategy, the European Commission made commitments relating to the publishing of negotiating mandates, textual proposals and the text of trade agreements as soon as these were negotiated (and prior to legal scrubbing) [55]. While the UK Government did show some movement on transparency in its response to the ITC report, its commitments in terms of publishing an outline approach and scoping assessment before negotiations commence, providing reports on each round before Parliament and publishing the final treaty text still fell short of EU transparency measures [58].

### Accountability

Accountability exists when one actor must explain their decisions and actions to specified others with the ability to mandate remedial actions and/or impose sanctions when necessary [38]. Clear lines of accountability are also key to establishing systems and actors within them who are responsible for identifying, and acting upon, health and equity impacts of trade policy, and that individuals and institutions are held to account regarding their actions in these areas. We identified two main issues concerning accountability; the role of the ITC and the proposed decision-making processes involved in trade remedies investigations. The primary role of the ITC is to scrutinise the work of the DIT, as summarised by the Committee’s Chair “As the October deadline approaches, and uncertainty mounts for businesses across the UK, my Committee will continue to hold the Department for International Trade to account …” [59] and accepted by the former Secretary of State; “As we begin to shape the UK’s independent trade policy and define our negotiating priorities and strategies, the International Trade Committee and Parliament will have an important part to play in holding us to account on our promise to secure the best possible outcomes for the whole of the UK” [60]. However, the strength of this commitment was called into doubt when a newly appointed Trade Secretary initially declined to attend the Committee for questioning [61].

During the period covered by our study, the ITC completed six inquiries, with a further six in progress. However, parliamentary Committees have limited powers, and while they can publish their concerns they cannot mandate that the Government demonstrates how they have taken Committee concerns or recommendations into account in guiding their future policies or actions. The ITC criticised the DIT for failing to provide information necessary to scrutinise its work [62], again serving to demonstrate the interconnectedness of governance elements, here between transparency and accountability. The Committee also raised concerns about accountability for ministerial decisions, as when it noted a lack of clarity about who was responsible, within Cabinet, for blocking investments where there were national security concerns [53].

A Trade Remedies Investigations Directorate (TRID) has been established within the DIT and, in due course, will transition into an arms-length body, the Trade Remedies Authority (TRA). The TRID is tasked with investigating “whether new trade remedies are needed to prevent injury to UK industries caused by unfair trading practices and unforeseen surges in imports … Our work helps to create a level playing-field for UK industries … Once the appropriate legislation is passed, TRID will become an arm’s length body – the Trade Remedies Authority” [63]. Such investigations, while vital to protecting domestic industry from unfair competition, often become highly contentious. The ITC took evidence from expert witnesses on the various ways in which such investigations are conducted elsewhere and the accountability structures that underpin them. Unlike many of them, the UK government has given final authority to the Secretary of State acting on the advice of the TRID. Greg Hands MP, the Minister of State rejected this view [64], arguing that ministers are accountable to Parliament. However, the ITC was concerned about a lack of clarity on how these decisions would be scrutinised and, if found to be wanting, would be sanctioned. Further concerns were raised by the ITC regarding the membership of the board of the TRA, with the ITC arguing for greater scrutiny of appointments and demonstrable independence from government, which it argued was essential to achieve credibility [65].

### Participation

Participation requires that affected parties are empowered with meaningful opportunities to access decision-makers in shaping policy [38, 66]. Participation is synergistic, but importantly distinct, from transparency, even though the two are sometimes conflated [66]. While transparency may allow various policy actors such as health or social justice advocates and the public to ‘see’ *who* is taking decisions, *how* and about *what*, participation, when conducted well, can facilitate more *active* involvement *within* the processes of deliberation and decision-making. It may therefore be seen as supporting a more democratic form of decision-making, facilitating critical and contrasting voices to be heard and for health and equity aspects of trade to be taken in consideration and acted upon in a timely manner. Furthermore, adequate participation of devolved nations/administrations and local governments is essential to ensuring the needs and interests of all parts of the UK are taken into account [37]. This is of particular relevance given that devolved nations/administrations and local governments hold competence for the delivery of health and social care, housing, education, and enforcement of environmental and food standards, for example, all of which can be impacted by trade agreements. It is also highly relevant given the government’s commitment that “we must have a transparent and inclusive future trade policy that delivers for all parts of the United Kingdom” [67]. Our analysis revealed many areas where participation is lacking. Here we focus on three specific areas: consultations, advisory or working groups, and the role of Parliament, devolved administrations and local government.

During the period that we analysed, the DIT held public consultations on future free trade agreements with the USA, New Zealand, Australia, and on potential accession to the Comprehensive and Progressive Agreement for Trans-Pacific Partnership (CPTPP). All four were launched online on 20 July 2018 and ran for 14 weeks (Fig. 1) [45]. The DIT has also held several outreach events, including Town Hall-style meetings and roundtable events and webinars. These had the stated goal of engaging a broad range of stakeholders [58]. Consultations were supported by summary documents [45]. Our analysis of these events and documents identified important contradictions. Consultations can only be meaningful if responses are used to guide future policy and actions. While respondents indicated their support for remaining aligned with the EU across many policy areas, and emphasised that securing a future economic partnership with the EU should be a priority, these messages did not appear in the DIT’s press release accompanying the publication of the findings. Subsequently, the UK government rejected alignment with the EU and advised companies that they will have to “adjust” to the new reality [68]. This is inconsistent with the written assurance by the Secretary of State to the chair of the ITC that “We have run one of the largest ever public consultations on potential free trade agreements … to ensure that the interests of businesses and individuals are considered during negotiation planning” [60]. It is difficult to avoid the conclusion that decisions are based on previously held, pro-Brexit positions, rather than the product of stakeholder consultation. Moreover, the government has since stated that it would not consult on the Future Economic Partnership (FEP) with the EU even though this involves a significant trade agreement. We were unable to identify the process by which this decision was made, a point which also relates to transparency.

It is also important to note that the consultation summaries were published on 18 July 2019, 9 months after the consultation processes closed, and far beyond the government’s commitment to report on such consultations within 12 weeks. This delay raises questions as to what would have happened if the UK had exited the EU in March 2019, as intended, as trade negotiations could then have started several months before the summaries had been published. Another concern, which also relates to transparency, is that individual responses to the consultations were not published. This contrasts with other consultations conducted by the UK government, a process that enables independent analysis of the views of stakeholders, identification of any concealed interests, and for assessing if and how the government is acting in accordance with the interests expressed by those consulted. Requests submitted by the first author to release them under Freedom of Information legislation have been denied.

As discussed under transparency, the STAG provides an opportunity to bring together a broad range of views. However, during the period of our analysis, the STAG had only met twice, in June and September 2019, according to publicly available records [43]. When established, it was stated that it would meet quarterly, with its membership reviewed annually. The ITC recognised that its formation was a step in the right direction but also expressed concerns about its membership, asking the government to “redress the imbalance between big business, small and medium business, civil society, trade unions and consumer groups” [58]. These concerns seem to have been shared by the members of STAG, with notes of the first meeting reporting questions about whether it was sufficiently representative of all stakeholder interests. The government also proposed that “The STAG will be complemented by a range of Expert Trade Advisory Groups (ETAGs) who will provide advice to the Government on specific sector and thematic policy issues. Their objective is to enable the Government to draw on external knowledge and experience to ensure that the UK’s trade policy is backed up by evidence at a detailed level and is able to deliver positive outcomes for the UK” [58]. In response to recommendations from the ITC that appointment to the STAG and sub-committees should be transparent and membership reviewed regularly, the Government stated that “Going forward, we are putting in place a continuous membership review process for the STAG and all ETAGs to ensure the groups fulfil their purpose and have a balanced representation” [58]. However, during our period of analysis, we were unable to identify any information on if, how and when ETAGs were to be established and their membership. It was not until July 2020 that Trade Advisory Groups (TAGs) were formed, with their membership being published on the 28th August 2020 [69]. Of note, there is a dominance of industry representatives, including from the alcohol and agri-food industries, with minimal, if any, health or social justice representation on each of the groups.

Beyond the opportunities for participation created by consultations and expert groups, Parliament has a key role, with members acting as representatives of the public. There have, however, been obvious tensions concerning the role of Parliament in scrutinising the government’s trade policy. The ITC recommended that “Parliament should be given an opportunity to debate the Government’s Outline Approach on a substantive motion before the mandate is set and negotiations commence” [58] and argued for a more robust system to scrutinise ratification of treaties, including free trade agreements, that would go beyond the existing mechanisms created by the Constitutional Reform and Governance Act (CRaG), which had been established when the UK was a member of the EU. The Committee also advised that “The House of Commons should have a final yes / no vote on the ratification of trade agreements” [58], reflecting the situation in the EU, where the European Parliament has a vote on all trade agreements since the Treaty of Lisbon [70]. In addition, where an EU trade agreement is considered to be ‘mixed’ (involving competences shared between the EU and Member States) it also requires ratification by national (and in some countries , sub-national) parliaments across the EU before it can fully come into force. Other legislatures cited in evidence to the ITC as having a vote on ratification of trade agreements include those in Australia, New Zealand and the US [55]. The government conceded that Parliamentary scrutiny was necessary but argued that existing mechanisms were sufficient [58]. The ITC also called for mechanisms to enable participation by the devolved nations, as is observed in some other countries, and local government, including a statutory intergovernmental international trade Committee for the devolved administrations and including participation from devolved and local governments on STAG. While the government formally welcomed these recommendations, we could not identify evidence to suggest that these mechanisms have been strengthened. Ministers did refer to a review of intergovernmental relations commissioned by the Joint Ministerial Committee, which brings together ministers from Westminster and the devolved administrations, but the review is still pending, with only draft principles for intergovernmental relations publicly available [71]. Further, the Internal Market Bill de facto dilutes the devolved competences of Northern Ireland, Scotland and Wales over a range of health matters [72]. Of note, McEwen and colleagues have documented the limitations of the current system, further exacerbated due to Brexit and the COVID-19 pandemic, calling for a radical overhaul of intergovernmental relations and decision-making processes [73]. Reference was also made to establishing a new inter-governmental Ministerial Forum for future trade agreements [67, 74], which might also offer a means for such consultations [75]. The inter-governmental Ministerial Forum for future trade agreements appears to have only met on a limited number of occasions. A first meeting scheduled for October 2019 was cancelled due to the upcoming General Election [76], with meetings since recorded in January and April 2020 [77, 78]. In contrast, evidence was presented to the ITC on the more institutionalised and extensive consultation of Canadian provincial governments in recent trade negotiations, including when setting the negotiating mandate and by integrating them into negotiating teams [55].

### Integrity

An organisation’s governance is said to have integrity when it has strong internal structures and processes underpinned by a mission and values that maintain high standards of conduct and prevent conflicts of interest and corruption [38]. Integrity is secured by the transparent allocation of roles and responsibilities, implementation of robust processes of decision-making, representation and enforcement, as well as adequate compensation and clear career paths that avoid ‘revolving-doors’ that can undermine it [17]. Integrity is about good management and has connotations with trust, which in turn can influence the degree and nature of stakeholder participation with important implications for inclusion of diverse groups and interests, including in relation to health and equity. When assessing an organisation’s integrity two important but distinct features need to be considered; are systems in place to promote integrity, *and* do they appear to be working? The ITC emphasised this point when it said that “Certain elements of consultation and oversight should be made statutory, to ensure that the system inspires the trust of stakeholders and mitigates concerns about unchecked executive power over trade policy” [55].

Integrity was also referred to by one expert witness from the perspective of its importance in building confidence and trust, among potential trading partners, in the processes by which the UK plans to undertake trade negotiations [55]. Wider reading demonstrated that there were significant concerns related to the matter of trust, with commentators pointing to failures to act on commitments already made in the Withdrawal Agreement, including measures to be taken regarding the Irish border [79].

Integrity involves adhering to commitments, including those on previously stated timelines. This was an area where the ITC raised many concerns. For example, in March 2019, the ITC asked why the membership of STAG had not been announced despite being assured by a DIT minister in November 2018 that it would be within that month. Similarly, on the 15th of March 2019, the ITC expressed alarm about an apparent lack of progress made in the rollover of EU free trade agreements, citing an analysis that demonstrated that the progress of many were at “code red”, despite previous assurances from the DIT that they were on track to meet the deadline of the 29th March 2019, the original Brexit date (Fig. 1) [80]. In an even stronger criticism, it said “In response to our report the Government wrote that the Government are committed to transparency, but it seems that the Government have actually consistently resisted putting transparency on some sort of statutory footing, despite acknowledging the importance of an open and inclusive trade policy. I understand that DIT has the worst performing record for responding to freedom of information. Why should we trust your commitments?” [81].

The ITC and its Chair questioned the mechanisms by which the DIT would ensure that its policies were aligned with the UK’s commitments to international development goals; “My Committee made a number of important recommendations in its report on this issue, and it is encouraging to see that in many cases, the Government is supportive. However, the vagueness of some of the responses doesn’t match the ambition needed to drive forward a trade policy that is fully aligned with development goals. The Government needs to commit to specifics – including timeframes that it can be held accountable to. Getting this right is important, not just for the UK but for the developing countries who rely on trade with us to support their development” [56].

Our findings should be placed in the context of perhaps the most important development related to integrity which arose late in the process in the latter half of 2020, when a UK minister conceded that the then Internal Market Bill, which as the Internal Market Act 2020 now governs relationships among the devolved four nations/administrations, would breach international law, albeit “in a very specific and limited way” [82]. This created shockwaves across the political spectrum, with all living former Prime Ministers speaking out against it. As has been widely noted, taken with its reluctance to agree with the EU on the level playing field, dispute settlement processes and other policies, for example on asylum seekers [83] and involvement of the armed forces in torture [84], this raises serious questions about the current UK Government’s commitment to international law, a fundamental marker of integrity.

### Capacity

Policy capacity often refers to the ability to develop policy that supports the achievement of clearly articulated goals with the resources at hand [38]. Evidence demonstrates that state capacity is key to realising some of the potential health benefits that can be gained from trade [85]. This is a particular concern given the unprecedented scale of work involved in transitioning to the UK’s new international trade policy arrangements and the lack of an apparent strategic direction, as noted by the ITC [86]. Of particular concern from a health and social justice perspective, is the capacity to identify, anticipate, mitigate and act upon trade aspects that hold implications for health and the interests of vulnerable groups. Our analysis revealed two important areas of focus: capacity within the DIT, and capacity within the UK more generally. In the first instance, the ITC noted how the UK was building up a negotiating capacity almost from scratch. The DIT has responded by creating what it describes as an International Trade Profession. The government’s Chief Trade Negotiation Adviser, who is also the Head of International Trade Profession, explained “we are ensuring government attracts the best and brightest talent by not only offering trade professionals a clear route into working on trade policy, exports and investment at the centre of government but also nurturing their ability for the future through access to world-class training” [87]. The DIT also plans to draw upon expertise through the STAG and the ETAGs (as discussed above). However, also noted above, there have been many concerns about the ability of the DIT to meet its commitments within agreed timeframes. The ITC has also raised concerns about the impact on capacity of cuts to the UK’s overseas representation and called on the government to dedicate appropriate resources to this area [53].

The ITC did, however, recognise that it was very difficult for the DIT to have recruited appropriate expertise given that, for most of the time during the Brexit negotiations, there was much uncertainty about the DIT’s future role and what would be required of its professional employees. Looking ahead, as one witness noted, “It is going to be enormously challenging to go through any of these negotiations, whether it be with the EU or the US or other large trading partners. The burden upon DIT is going to be enormous. Whatever process is set up has to be as sensible as possible. There has to be an appropriate balance between consultation and getting the work done because you do not have an infinite number of people to do this” [57]. This clearly demonstrates the interplay between capacity and participation, with capacity constraints potentially impacting on the ability to enable meaningful participation by different groups.

Finally, it was noted by the ITC and expert witnesses that it will be necessary for those involved in international trade, including all tiers of government, business, and others, to have the necessary expertise and understanding. However, achieving this will require additional resources and support, particularly at the local level [57].

## Discussion

Countries regularly engage in negotiations on international trade and investment, either individually or as members of trading blocs. These negotiations involve decisions that have implications for health [14]. Yet there are concerns that health, where it is discussed at all, is often low on agendas or narrow in scope [18]. Those responsible for establishing trade governance systems in the past might be forgiven for overlooking mechanisms by which health concerns can be raised. An expanded body of research on the links between international trade and health in the past decade means that this is no longer justifiable. The UK’s post-Brexit trade policy agenda has far-reaching implications for health and social justice, and strong systems of governance are needed to enable inclusion of health and equity considerations, and ensure commitments to these agendas are met. Our analysis suggests that UK trade policy governance is far from robust, with significant implications for health and social justice.

At first glance, our analysis indicates that the elements required for good governance might appear to have been implemented or proposed. For example, a large volume of information has been placed in the public domain. The government has undertaken public consultations on its proposed trade negotiations. Ministers remain accountable to Parliament, although less so than before as a result of changes to the EU Withdrawal Bill introduced by the Johnson administration, and the civil service is strengthening its capacity to engage in negotiations on international trade. However, our detailed examination of the work of the DIT and ITC identifies numerous weaknesses. The ITC reported that the DIT was at times insufficiently transparent to permit adequate scrutiny. The Committee struggled to understand the lines of accountability involved in future trade agreements, especially when they involved other areas of government, such as security and development. Reporting on public consultations and market research appeared to be based on selective interpretation, while key actors, such as the devolved administrations and local government, who are crucial in the delivery of services and enforcement of standards that are critical to health and equity, were largely excluded and mechanisms for their ongoing participation remain underdeveloped. Lack of clarity about the operation of the DIT, coupled with repeated failure to meet deadlines led the ITC, exceptionally, to ask whether the Government’s commitments could be trusted, thereby questioning its integrity. Building sufficient capacity in a complex area, from a standing start, was always going to be difficult and much has been achieved by the DIT since the Brexit referendum. However, there is clearly much more progress needed to strengthen trade policy governance to minimise the risk of poor policy development and negative outcomes, including unintended or inequitable distribution of harms.

We approach this examination of the governance of trade using a public health and social justice lens. However, in marked contrast to much of the media discourse, with its focus on food safety and protecting the NHS, it was notable that health was rarely mentioned in the texts we analysed. This is perhaps not surprising, as the priority has been to put in place a system that can take forward the UK’s new international trade agenda, scrutinised by MPs through the ITC. However, there is no guarantee that issues involving health such as environmental and worker safety standards will be addressed, or even how health and equity considerations might be incorporated within trade negotiations. It has been noted previously how health and equity remain largely absent from trade and investment policy agendas, due in part to fundamental institutional and ideological differences, with trade policy-making being predominantly driven by neoliberal ideas of export growth, private enterprise and reduced regulation, which limits consideration of wider determinants of health [14, 28, 88]. Notably, despite decades of mounting evidence on the health and equity impacts of trade and the role of strong systems of governance in mitigating avoidable harms from trade [8, 14, 17, 89], the UK government has largely ignored this evidence and failed to galvanise the opportunity to include public health and equity considerations and strengthen democratic involvement in trade policy. This underscores the point that the evidence alone will not guarantee that health and justice are prioritised, and has implications for social justice and public health advocates [28, 90]. It also highlights the need for greater advocacy on broadening the scope of the debate which all too often adopts a narrow problematisation of the impact of trade on health, prominent examples being a focus on specific *products* such as chlorinated chicken or, in the context of the COVID-19 pandemic, direct *processes* such as supply chains [91]. Despite repeated calls by health advocates to take the complex interplay between trade and the wider determinants of health and equity more seriously, these issues have all too often received little or no attention.

Negotiations undertaken within the framework of the WTO and many regional groupings, such as the Association of Southeast Asian Nations (ASEAN) or the Southern Common Market (MERCOSUR), are largely unconstrained by health considerations. A rare exception is trade in tobacco, where the parties involved can draw on the provisions of the Framework Convention on Tobacco Control. This is different from the situation within the EU Single Market, with the European treaties stating that a high level of human health shall form part of the EU’s policies – even if some have criticised the subordination of health policy to neoliberal market logics [92]. Crucially, this requirement is powerful, as it must be taken into account in decisions of the European Court of Justice. It was notable that the British Government refused to enshrine its commitment to “do no harm” as it withdrew from the EU, despite calls to do so [93]. As a consequence, the British public will lack the legal protection of their health that they held as EU citizens. Furthermore, interests of UK citizens and residents will no longer directly benefit from the actions of public health advocacy organisations that are based in Brussels and have years of experience in lobbying the European Commission, including on trade-related issues [94, 95]. In light of these considerations, it is concerning that the UK government appears to have reduced not strengthened the systems of trade governance, despite the opportunity to do the latter upon establishing the DIT and despite receiving detailed evidence-based recommendations on how to do so by the ITC, including in respect of formalising consultation mechanisms. The centralised nature of the UK state, with the Royal Prerogative covering treaty-making (including trade and investment agreements), and the consequent limited Constitutionally-enshrined role for Parliament, devolved administrations and local government [37], represents a potential problem for participation and transparency in trade governance. The selective interpretation of the consultation response is also of concern as is the lack of advanced mechanisms through which to promote meaningful participation of devolved and local governments. Indeed, the House of Lords EU Select Committee has highlighted the lack of consultation with the devolved nations/administrations; draft texts of roll over agreements were not shared prior to signature, which is described as “puzzling and potentially damaging” [96].

A further issue concerns the potential constraint future trade agreements may place on the opportunity for regulatory experimentation that the UK could otherwise benefit from upon leaving the EU. In the event the four nations/administrations are no longer constrained by EU standards, diversity in regulation between them (for example, with regards to alcohol policy) could be an opportunity for public health policy to be informed by policy learning. However, future trade negotiations and agreements, largely driven by centralised decision-making, could constrain the public health policy decisions of the devolved nations/administrations, hindering the development of diverse approaches and associated policy learning. This is what appears to be intended by the Internal Market Act 2020.

### Strengths and limitations

This study is the first, to our knowledge, to look in detail at the emerging system of trade policy governance in the UK following Brexit, and consider its effects on health and social justice. It has benefited from the often forensic questioning and investigation by members of the ITC, but it is also constrained by being dependent on information that is in the public domain, as requests for additional data under freedom of information legislation were declined.

We did not examine the arrangements governing the negotiation of a trade agreement with the EU. As has been described at length elsewhere, this is an area where the UK Government sought to maintain a high level of secrecy, in marked contrast to the move towards greater transparency that has characterised the EU – a shift that occurred in part because of coordinated advocacy efforts across the EU, including by groups in the UK [94, 97]. Early in the process of negotiating a new trade agreement, the EU published a 350 page draft text of a future agreement, with Michel Barnier (negotiator for the EU) observing at the time the UK Government’s refusal to put any of its corresponding text into the public domain. Although not part of our analysis here, the EU-UK Trade and Cooperation Agreement as agreed in late December 2020 has major omissions when it comes to health and social justice protection [98].

We have conducted an initial documentary analysis, exploring all aspects of governance captured within the TAPIC framework. A next step would involve going beyond the documents to undertake interviews with key actors, within and beyond government to build on the current study which looks at *what* has been established but is unable to explore *why* or *how*. In particular, it will be important to look at how trade negotiations are informed by other government departments that may have relevant competencies, such as the Department of Health and Social Care, the Foreign, Commonwealth and Development Office and the Department for Environment, Food, and Rural Affairs, some of which may have competing interests. For example, those negotiating provisions on pharmaceuticals must balance health policy, with the imperative of keeping down costs, and economic policy, which will seek to support the profitability of British pharmaceutical companies.

Our analysis was also restricted to trade policy governance using the TAPIC framework. Other aspects of (potential) policy failure need to be researched. Efforts aimed at identifying why policies have failed, or what risks of failure exist, and how to strengthen policy design and implementation, need to consider “is this a governance problem or is there another explanation?” [38]. For example, the problem may be a lack of funding and/or political will [38]. While we have identified a number of governance issues, the extent to which these other influences may also potentially compromise successful realisation of trade benefits, particularly from a health and equity perspective, needs to be established. Additional and important areas for consideration include the extent to which the neoliberal ideas that guide international trade and investment agreements and the associated privileging of commercial interests preclude efforts to advance health and justice [28]. Such changes will need to be accompanied by more extensive and robust carve-outs that recognise the specific characteristics of health and social services, a review of intellectual property rights provisions that undermine access to medicines, as well as shifts in normative assumptions about the legitimacy of trade agreements that undermine health and social justice [15, 18]. Such matters, however, also underscore the wider significance and effects of addressing matters of trade governance. Tackling them requires challenging the long-standing “deliberative exclusion” of non-trade concerns and public interest groups from technocratic and opaque decision-making processes [99].

While we focused exclusively on the governance of trade policy, further research is also needed to evaluate the governance of other policy spaces in the UK during this time of radical policy transformation which has profound implications for health. There is growing concern about the current government’s commitment to systems of governance and democracy. For example, the Institute for Government’s 2020 Whitehall Monitor report on government transparency raised alarms about the government’s poor adherence to freedom of information requests, and concerns have been raised about governmental departments failing to issue spending reports on a regular basis, and regarding the decision to change the location of parliamentary briefings to Downing Street from the House of Commons which has been described as a threat to media access and limits the use of mobile phones [100, 101]. Our findings also call into question how the governance deficits identified compromise the UK’s fulfilment of the sustainable development goals (SDGs), such as SDG 16; “Promote peaceful and inclusive societies for sustainable development, provide access to justice for all and build effective, accountable and inclusive institutions at all levels” [102], and requires further investigation. Furthermore, international organisations, such as the WTO, recognise that trade should be used as a vehicle to achieve other SDGs [103], whereas this analysis suggests that the way negotiations are being governed in the UK could compromise any such opportunities, insofar as they exist. More broadly, the lack of investment in trade policy governance and failure to create systems that prioritise health and social justice considerations, lies in direct contrast to the growing recognition among global health actors for there to be greater coherence between environmental, social and economic governance, which should no longer be pursued along divergent paths [8].

Finally, as trade negotiations are in the early stages, it is too early to know whose and what interests will be prioritised, including health and equity considerations. So far, apart from the EU-UK Trade and Cooperation Agreement, the agreements that have been reached have been mostly replicating the agreements that already existed with the EU. Going forward, it will be necessary to subject them to scrutiny, with academia and civil society likely playing an important role as has been the case historically with trade negotiations [94, 97].

## Conclusions

Future trade and investment agreements have profound implications for life in the UK. They will influence the food that is available, the conditions in which it was produced, the information that will allow consumers to make healthy choices, product marketing and how this is regulated, the price paid for medicines, and much else. They have the potential to constrain the policy tools needed to meet future health and environmental challenges. Norms and values well established in the UK, and embedded into such policies as safety regulations, could be abandoned or watered down, instead labelled as non-tariff barriers to trade.

The question of post-Brexit governance arrangements also points to the importance of rallying health and social justice advocacy communities so that, at this moment of change, health and equity considerations form part of the new trade policy landscape. While there need to be appropriate openings in governance structures, which advocates should also push for themselves, these then also need to be seized; the evidence regarding the relationship between trade, health, and social justice does not just speak for itself, and our findings demonstrate that there is much advocacy work needed to ensure health and equity inform UK policy decisions. This point also resonates beyond the UK, with the COVID-19 pandemic representing a significant critical juncture and opportunity for progressive change. While it has raised the profile of health in a trade policy context, being linked to the operation of global supply chains for medical goods, what we need is a more holistic understanding of the way in which trade and health interact [104]. At a time where there is a risk that the COVID-19 pandemic will lead to a more enduring concentration of decision-making power in the hands of states [105], the questions raised by the TAPIC governance framework remain as relevant as ever.

## Methods

We conducted a qualitative analysis of UK trade policy governance using the TAPIC framework [38]. This framework, informed by a review of the international literature on governance, was initially developed by a team of researchers to evaluate governance in the health sector and has been applied to a growing number of issues in Europe and North America, including trade policy [17, 106–108]. TAPIC is an acronym for Transparency, Accountability, Participation, Integrity, and policy Capacity (Table 1). Weaknesses in any one of these characteristics increases the risk of poor policy, risks to health, and foregone opportunities for health gain, particularly in relation to design, implementation, and outcomes. Overall, weaknesses in governance can be attributed to one or more of the TAPIC elements, largely due to issues of excess, inadequacy, or inappropriate application of a governance tool [38]. Conversely, strong systems of governance can serve to ensure different voices, forms of evidence, and perspectives, such as health and social justice, are heard and taken into consideration, and for potential negative or unintended consequences to be identified, mitigated, or addressed, and beneficial outcomes realised and equitably distributed. We applied this framework using a public health lens, in that we assume that trade negotiations should protect health and, ideally enhance it, while taking a normative view that, where there are trade-offs, groups that are already vulnerable or disadvantaged should be protected. This is consistent with the government’s stated commitment to “do no harm” during the Brexit process [109], and its wider electoral commitment “to unite and level up, spreading opportunity across the whole United Kingdom” [110].

**Table 1:** The TAPIC framework elements (adapted from Greer 2016, 2017, Jarman 2017)

TAPIC element	Definition	Relevance to health and health equity	Example mechanism	Example challenges
Transparency	An institution is said to be transparent when decisions, and the processes and grounds on which decisions are made, can be observed and the public and other relevant actors are informed about them. Transparency is more than simply recording activities but requires that the information about them is readily accessible, accurate, timely, and comprehensive, and is presented in a way that is comprehensible, taking account of the technical issues that might arise.	Transparency is central to empowering members of health, environmental and social justice communities including health professionals, civil society and researchers, for example, to understand *how* decisions are being made, *what* is being considered and *why*, and to identity potential impacts	Information provision and open document publication, Transparency / lobbying / interests registers, Freedom of information requests, External audit	Countering concerns that transparency will translate to increased public criticism, weakening of negotiating positions, or failed trade agreements Conflicts between national transparency agenda and level of transparency required by trading partners
Accountability	Accountability exists when one actor must explain their decisions and actions to specified others with the ability to mandate remedial actions and/or impose sanctions when necessary.	Clear lines of accountability are key to establishing systems and actors within them who are responsible for identifying, and acting upon, health and equity impacts of trade policy, and to ensure that individuals and institutions are held to account regarding their actions in these areas.	Scrutiny committees, Legislative mandates, Trade impact assessments, Transnational arbitration/dispute settlement mechanism	Legislative scrutiny may be reduced when decision-making power is delegated to executive agencies Large scale and rapid institutional change can make lines of accountability less clear Trade impact assessments may not include health and equity considerations Dispute settlement prioritise economic norms
Participation	Participation requires that affected parties are empowered with meaningful opportunities to access decision-makers in shaping policy.	While transparency may allow various policy actors such as health or social justice advocates and the public to ‘see’ *who* is taking decisions, *how* and about *what*, participation, when conducted well, can facilitate more *active* involvement *within* the processes of deliberation and decision-making. It may therefore be seen as supporting a more democratic form of decision-making, facilitating critical and contrasting voices to be heard and for health and equity aspects of trade to be taken in consideration and acted upon in a timely manner.	Public consultation, Public forums / webinars, Trade advisory committees	Overcoming knowledge barriers to participation. Restrictive membership of advisory committees. Public consultations and dialogues may not be routinized or inclusive enough
Integrity	An organisation is said to have integrity when it has strong internal systems and rules, underpinned by missions and cultures that also promote integrity.	Integrity is about good management and has connotations with trust, which in turn can influence the degree and nature of stakeholder participation with important implications for inclusion of diverse groups and interests, including in relation to health and equity.	Risk registries, Internal career path, International laws, treaties, commitments (e.g. Framework Convention on Tobacco Control, Doha Declaration, Sustainable Development Goals)	Codes of conduct are often advisory in nature Revolving doors for policy-makers exist between government and industry
Capacity	Policy capacity often refers to the ability to develop policy that supports the achievement of desired goals with the resources at hand.	Of particular concern from a health and social justice perspective, is the capacity to identify, anticipate, mitigate and act upon trade aspects that hold implications for health and the interests of vulnerable groups.	Trade-specific capacity building, Training of health staff and vice versa in trade policy (e.g. WTO online training modules)	Exit from EU and loss of capacity Complexity of trade deals and their impacts Difficulty of inter-departmental working Trade staff are not health specialists

Our starting point was the reports and proceedings involving the DIT and the ITC in the period from the inception of the DIT and ITC to the Dissolution of Parliament on 6th November 2019 ahead of the UK General Election on 12th December 2019. We use the latter date as a cut-off as the UK left the EU on 31st January 2020 and the ITC has only published one report in the intervening period (until November 2020), on the implications of the COVID-19 pandemic, which says little about trade policy governance [111]. In total, we identified 416 relevant documents from varied sources, as follows (Table 2). First, all publications from the News andNews and communications sections of the ITC and DIT websites respectively were extracted and collated. Second, all other sections of the DIT website were checked for material relevant to trade governance, such as minutes of advisory group meetings, performance dashboards, and consultation documents. Third, we reviewed the transcripts of oral evidence sessions and final reports for all completed inquires held by the ITC and, where available, the Government’s response. Fourth, we used a snowballing technique to follow up references to other reports or webpages cited within the material reviewed. At the same time, we read widely on Brexit and trade related issues to place the findings in a wider context.

**Table 2:** Sources, number and types of documents comprising the study dataset

Source	Section within source	Number of items / documents	Types of documents
Department of International Trade	News and communications	33	Press releases, speeches, events, announcements
	Policy papers and consultations	131	Consultation support documents, policy documents
	Research and statistics	33	Market research reports, annual reports
	Transparency and Freedom of Information Releases	17	Freedom of Information Releases
	Our Governance	3	Department structure, interest registries, terms of references
International Trade Committee	News	90	Inquiry announcements, news / press releases, inquiry updates
	Inquiries	109	Oral session transcripts, committee reports, government responses

Whilst we sought to ensure that we had a dataset that was comprehensive and relevant, it was also necessary to ensure that it was manageable. We therefore decided to focus on the work of the ITC which was established to directly scrutinise the activities and running of the DIT. Thus, we excluded material from enquiries related to the work of the DIT conducted by other House of Commons and House of Lords Committees, and data related to the DIT’s involvement in some of its inherited responsibilities, the Prosperity Fund (involving international development), the Conflict, Stability, and Security Fund programs, and the Sanctions and Anti-Money Laundering Bill, as the governance of these cross-departmental initiatives lay outside the scope of the analysis. We also excluded the ITC’s work with three other Commons Committees in the context of the Committees on Arms Export Controls. Written submissions to ITC inquiries were also excluded, again to maintain manageability and because the content of these submissions inform oral sessions and final reports by the ITC, which were included in the dataset. However, to ensure the exclusion of these documents did not impact our findings we conducted a brief review of a selection of excluded documents. We did not identify any obvious additions to the general governance aspects identified through the data sources that were included.

We applied a deductive approach to coding the material, using the five elements of the TAPIC framework as a pre-specified thematic framework to code the data, which is appropriate for structured analyses of pre-defined policy processes [112]. Using a framework approach, we proceeded by means of familiarisation, indexing, charting, and mapping and interpretation [112]. After initial coding of the majority of the data by MvS, a purposive sample that included different types of documents and governance issues, was extracted and coded independently by PB to ensure that there was a consensus on the assignment of data to each element.

The coded data enabled a systematic and in-depth analysis of the workings of the DIT and ITC and elaboration of the TAPIC elements. We identified main issues concerning each of the elements, and reviewed whether what was reported potentially strengthened or weakened governance arrangements, again as seen through a public health lens. Where we were uncertain, we supplemented our analysis by consulting the literature on governance and discussion among all authors.

## Data Availability

All data used for the research is publicly available.

## Footnotes

1 Investor-to-State Dispute Settlement, or ISDS, is a procedural mechanism via which a foreign investor can initiate arbitral proceedings against a state for certain violations of investor rights spelled out in an IIA. Health policies have been challenged under ISDS proceedings, e.g. a plain packaging law for tobacco products in the *Philip Morris v. Australia* case.
